# Oral Health Promotion Among Students in Special Education Schools: Protocol for a Multicity Cluster Randomized Controlled Trial

**DOI:** 10.2196/93889

**Published:** 2026-07-03

**Authors:** Ruihang Zhang, Rong Lin, Wenyan Huang, Sujuan Zeng, Jiao Wang, Si Meng, Lin Xu

**Affiliations:** 1 School of Public Health, Sun Yat-sen University, 74 Zhongshan 2nd Road, Guangzhou, Guangdong, China; 2 Greater Bay Area Public Health Research Collaboration, Guangdong, China; 3 Guangzhou Center for Disease Control and Prevention (Guangzhou Health Supervision Institute), Guangzhou, China; 4 Department of Pediatric dentistry, School and Hospital of Stomatology, Guangdong Engineering Research Center of Oral Restoration and Reconstruction & Guangzhou Key Laboratory of Basic and Applied Research of Oral Regenerative Medicine, Guangzhou Medical University, Guangzhou, China; 5 School of Public Health, University of Hong Kong, Hong Kong, China (Hong Kong); 6 Department of Applied Health Sciences, University of Birmingham, Birmingham, B15 2TT, United Kingdom, 44 121 414 3344

**Keywords:** oral health, special education schools, students with disabilities, fluoride varnish, dental caries, cluster randomized controlled trial

## Abstract

**Background:**

Children with disabilities attending special education schools face significantly higher risks of oral diseases, particularly dental caries, due to physiological, cognitive, and environmental challenges. However, school-based oral health interventions targeting this population are limited in China.

**Objective:**

This study aims to assess the effectiveness of fluoride varnish (FV) application, delivered alongside oral health education for parents and teachers, in improving the oral health of students in special education schools in Guangdong Province.

**Methods:**

This is a stratified, cluster randomized controlled trial conducted in Guangdong Province, China. Schools will be stratified by regional economic development level and randomly assigned to either an intervention or control group. The intervention group will receive annual dental examinations, biannual FV applications, and oral health education. The control group will receive standard annual examinations and comparable oral health education. Participants will be students aged 5 to 22 years enrolled in selected special education schools. Baseline assessments and 12-month follow-ups will include oral examinations and questionnaires assessing oral health knowledge, attitudes, and behaviors. The primary outcome is the difference in caries prevalence between the intervention and control groups at follow-up. Secondary outcomes include changes in oral hygiene behaviors and caregiver knowledge. Data will be analyzed using multivariable regression models adjusting for potential confounders and clustering effects.

**Results:**

This trial was funded by the Department of Education of Guangdong Province in 2025 (grant YCKJ-2025-30). As of June 2025, 12 special education schools across 3 cities had been enrolled (n=7, 58.3% intervention and n=5, 41.7% control schools), and more than 1100 students had been recruited. Baseline data collection and the first FV application are complete. Follow-up data collection is ongoing and is expected to conclude in June 2026. Data analysis has not yet started, and results are expected to be published in 2027.

**Conclusions:**

Biannual application of FV is expected to significantly reduce caries prevalence and improve oral hygiene behaviors among students in special education schools. This school-based intervention model aims to provide a scalable and evidence-based strategy to mitigate oral health inequities for children with disabilities in China.

## Introduction

Oral health is a critical component of general health and quality of life, yet children with disabilities remain an underserved population with significantly elevated risk for oral diseases [1,2]. Studies across multiple settings have consistently shown that students with disabilities experience a higher prevalence and severity of dental caries, gingival inflammation, and oral hygiene problems than their peers without disabilities [3]. This disparity is attributed to a range of factors, including motor or cognitive impairments, reduced self-care abilities, dependence on caregivers, behavioral challenges, and limited access to dental services [3,4]. In China, these challenges are compounded by underdeveloped oral health support systems within special education settings, despite national goals to promote equitable health access for all populations [5].

Fluoride varnish (FV) is widely endorsed as an effective caries-preventive intervention, particularly in children at moderate to high risk [6,7]. Meta-analyses, including multiple Cochrane reviews encompassing more than 200 trials and 80,000 participants, have consistently reported significant reductions in caries incidence, up to 43% in permanent teeth and 37% in primary teeth, with biannual professional FV application [6-9]. Additional studies have demonstrated its efficacy in preventing white spot lesions in adolescents with orthodontic appliances and reducing caries in special-needs populations, such as children with attention-deficit/hyperactivity disorder [10,11]. However, the overall body of evidence remains inconclusive. Some more recent systematic reviews and large-scale trials have shown limited or no significant benefit, particularly in low-risk populations or settings with high background fluoride exposure [7,12]. Furthermore, substantial heterogeneity across studies, limited methodological quality in earlier trials, and uncertainty in comparative effectiveness vs other preventive interventions (eg, sealants) complicate interpretation [9,13]. This conflicting evidence underscores the need for rigorously designed, context-specific evaluations of FV effectiveness, particularly in high-risk, underserved populations, such as children with disabilities in school-based settings.

To date, few randomized trials have evaluated FV as a standalone intervention in students attending special education schools, especially in low- and middle-income countries [3,11]. Existing research often combines FV with other interventions, targets only certain subtypes of disabilities (eg, intellectual disability), or lacks sufficient sample sizes and follow-up to assess effectiveness under real-world conditions [11]. In China, there is currently no trial evaluating the effect of FV in special education settings, nor any that incorporate systematic delivery through existing school health infrastructure.

This study addresses this critical evidence gap by evaluating a school-based FV intervention in special education schools in Guangdong Province, China, using a stratified cluster randomized controlled trial (RCT) design. The trial compares the effect of biannual FV applications, administered by trained professionals and accompanied by tailored oral health education for parents and teachers, with that of standard care alone. By assessing the impact of FV in a high-risk, underserved population, this study seeks to generate policy-relevant evidence to inform national oral health strategies for children with disabilities.

## Methods

### Study Design

This study is a stratified cluster RCT designed to evaluate the effectiveness of FV in preventing dental caries among students attending special education schools in Guangdong Province, China. This trial is open label. Neither participants, caregivers, nor outcome assessors will be blinded to group allocation due to the nature of the intervention.

### Setting

All special education schools located in Guangdong Province are eligible for inclusion if they provide primary and/or secondary education to students with disabilities. No minimum enrollment threshold is required, and schools are not excluded based on size or student composition. All participating institutions are formally registered educational entities operating under the supervision of local education authorities. According to the Guangdong Provincial Education Bureau, there are a total of 162 special education schools distributed across 21 prefecture-level cities. Cities such as Guangzhou (n=19, 11.7%), Shenzhen (n=11, 6.8%), and Shantou (n=8, 4.9%) have relatively higher concentrations of eligible schools. The sampling frame for this trial was constructed using the official registry of special education schools.

### Stratification, Randomization, and Allocation Concealment

**Figure 1.**
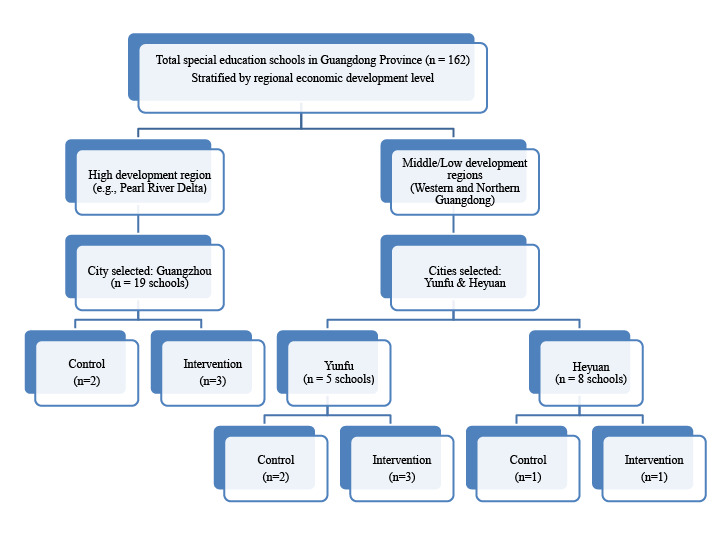
Flow diagram of school selection for the cluster randomized controlled trial.

Guangzhou, representing a high economic development region within the Pearl River Delta along with Yunfu and Heyuan, which represent middle-to-low development regions from western and northern Guangdong, respectively, were purposively selected to ensure geographic and socioeconomic diversity. Within each of these 3 cities, eligible schools were identified from lists of schools willing to participate provided by local education authorities. School-level cluster randomization was then performed stratified by city. A computer-generated random allocation sequence was produced by an independent statistician who was not involved in school recruitment, baseline assessment, or intervention implementation. Schools within each city were randomly assigned to either the intervention or control group according to this sequence. To ensure allocation concealment, opaque, sealed envelopes containing group assignments were prepared by staff not involved in intervention implementation. Allocations will be disclosed only after completion of school enrollment. Ultimately, 5 control schools and 7 intervention schools were selected across the 3 cities to participate in the study (Figure 1).

### Inclusion and Exclusion Criteria

Students enrolled in participating schools will be recruited, with informed consent obtained from their legal guardians.

Students will be excluded if they have bronchial asthma, a known severe allergy or hypersensitivity to FV. They will also be excluded if they have acute oral conditions or severe systemic diseases. Students who are unable to cooperate or refuse to participate in the study will also be excluded.

### Sample Size

The primary outcome of this study is the prevalence of dental caries at 12 months. On the basis of previous literature in comparable populations, the prevalence of caries among students in special education schools without intervention is estimated at 60% [5,14]. A reduction to 40% in the intervention group is considered clinically and politically meaningful [9,15]. Using a 2-sided α of .05 and a power of 90% (β=.10), the required sample size per group for an individually randomized trial is estimated using the formula:


$$n=\frac{{\left(z_{\alpha /2}+Z_{\beta }\right)}^{2}\times \left(p_{1}(1-p_{1})+p_{2}(1-p_{2})\right)}{{\left(p_{1}-p_{2}\right)}^{2}}$$

where *z*_*α*/2_ is 1.96, *Z*_*β*_ is 1.28, *p*_1_ is 0.60, and *p*_2_ is 0.40. Substituting these values gives a sample size of approximately 126 per group under individual randomization. Additionally, this study uses a stratified cluster RCT design, which requires adjustment for clustering using the design effect. The design effect was calculated as 1+(m–1)×*ρ* where *m*=150 (average cluster size, ie, school size), *ρ* is 0.02 (intracluster correlation coefficient). The resulting design effect was calculated as 3.98, yielding an adjusted sample size of 501 students per group. Allowing for an anticipated 10% dropout rate, the final required sample size is 557 students per group, totaling 1114 participants across all clusters. On the basis of the final required sample size of 557 students per group and an average cluster size of 150 students per school, the required number of clusters was calculated as 557/150=3.71 per group. Therefore, at least 4 schools per group, corresponding to a minimum of 8 clusters in total, are required.

### Intervention and Control

The intervention group will receive 2 professionally administered FV applications. Applications will occur at baseline (month 0) and at 6 months, using Duraphat (Colgate-Palmolive Company) varnish, which contains 5% sodium fluoride by weight, equivalent to 22,600 parts per million fluoride ions [16,17]. The varnish will be applied to all erupted tooth surfaces by trained dental professionals under standard infection control procedures, including hand hygiene, appropriate personal protective equipment, and the use of single-use disposable materials to prevent cross-contamination, in accordance with Centers for Disease Control and Prevention guidelines for portable dental care [18]. A brief oral examination will be conducted prior to each session to confirm eligibility for varnish application. Prior to application, the teeth will be cleaned with sterile gauze and isolated with cotton rolls for moisture control [19]. The tooth surfaces will then be dried with gauze, followed by the application of 0.40 mL of 5% sodium fluoride varnish [20]. Concurrently, oral health education will be provided to parents and teachers of students. These educational sessions will be delivered during the school visits in which varnish applications occur. Content will include basic oral hygiene practices, the importance of fluoride in caries prevention, dietary advice, and strategies for supporting children with disabilities in maintaining oral health.

**Figure 2.**
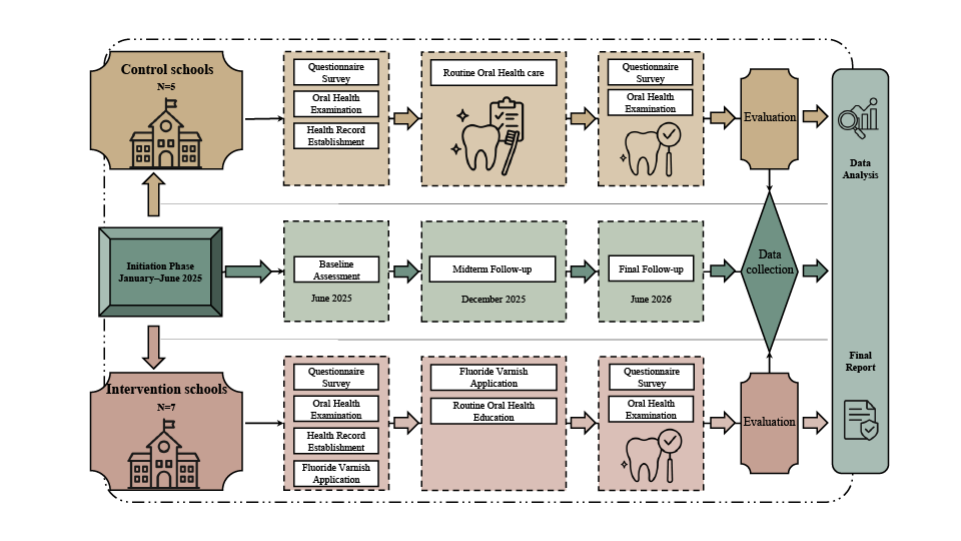
Overview of the study design and timeline for the fluoride varnish intervention in special education schools in Guangdong Province from 2025 to 2026.

The control group will receive standard care, consisting of annual oral health examinations and oral health education. These educational components will be delivered during routine school visits using the same materials and format as in the intervention group, ensuring comparability of educational exposure between groups. However, no FV will be applied in control schools during the study period. The overview of the study is shown in Figure 2.

### Study Outcomes

**Figure 3.**
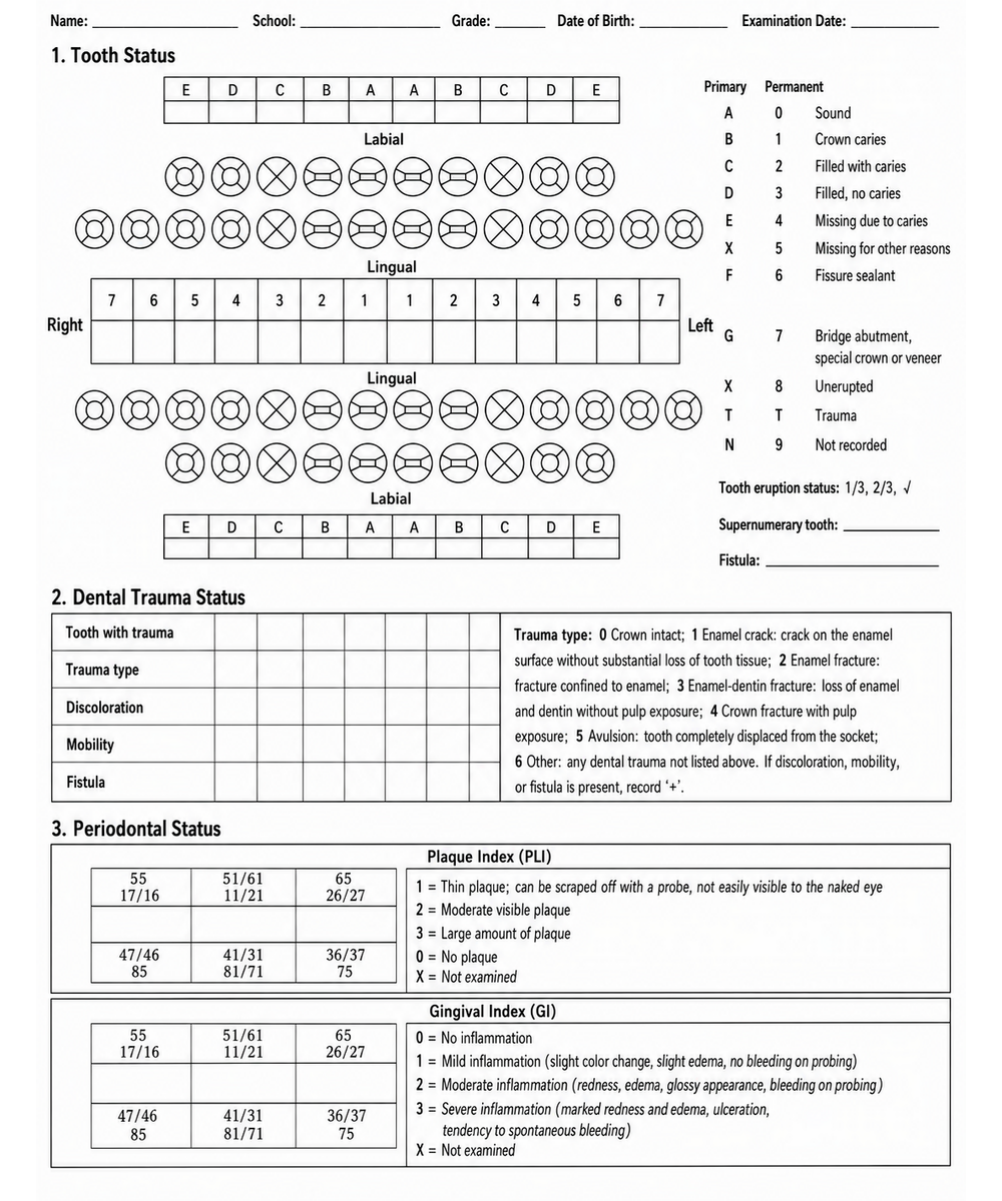
Blank recording form for oral examination.

The primary outcome of this study is the prevalence of dental caries among students at 12 months following the intervention. Dental caries will be assessed through clinical oral examinations using the decayed, missing, and filled teeth index (DMFT) for permanent and primary dentition, respectively. These examinations will be conducted in accordance with the national standardized child oral health survey protocol used in China. Briefly, trained and calibrated dental professionals will perform tooth-level examinations using disposable dental mirrors and probes under natural or portable lighting. Dental status for both primary and permanent teeth, including caries experience and related conditions, will be recorded. Examinations will take place in a designated room with adequate lighting [21]. Children will be assessed while seated [22], and wheelchair users will remain in their own wheelchairs. Two familiar teachers or school support staff will attend to assist with communication and comfort [23]. A trained dentist will conduct the examination, and a trained nurse will record the number of decayed, missing due to caries, and filled teeth per child. The blank recording form is provided in Figure 3. To minimize measurement bias, examiners conducting the follow-up assessments will be blinded to the school allocation status.

Secondary outcomes include (1) oral hygiene status (eg, presence of plaque and gingivitis), assessed using gingival health indicators and plaque indices; (2) changes in oral health–related knowledge, attitudes, and practices (KAP) among students, parents, and teachers, measured using a structured questionnaire adapted from the Third National Oral Health Survey [24].

Baseline assessments will be conducted prior to intervention implementation, with follow-up occurring 12 months later. Oral examinations will be performed on-site in schools, with each child examined in a standardized manner. KAP questionnaires will be administered to parents and teachers at both baseline and the end of follow-up. All data will be collected using the Wenjuan Star platform (Shanghai Zhongyan Network Technology Co Ltd) or through paper-based questionnaires with double data entry, depending on the logistical feasibility at each study site. Data from paper questionnaires will be manually entered into the electronic system by trained students at the university. Details of the information collected are shown in Table 1. Monitors appointed by the trial sponsor will oversee trial implementation to ensure protocol compliance and data accuracy (Multimedia Appendix 1).

**Table 1.** Baseline information collected via a questionnaire and oral health examination.

Domains and variables	Units and categories
Demographics	
Sex	Male; female
Age	Years
Ethnicity	Han; other
Height	Centimeters
Weight	Kilograms
School	School name
Grade	Primary; middle; high
Class	Class name or number
Socioeconomic information	
Parent’s education level	Junior high; high school; college; postgraduate
Parent’s occupation	Government; private; other
Perceived dental cost burden	Very cheap to very expensive
Caregiving context	
Relationship to child	Father; mother; grandparent; other
Primary caregiver	Parent; relative; nanny; other
Boarding status	Yes; no
Dietary habits	
Sweet food or drink preferences	Yes; No
Use of sweets as rewards	Never; sometimes; often; unknown
Night-time snack intake	Never; sometimes; often; unknown
Daily sugar intake frequency	≥3 times/day: yes; no; unknown
Tooth brushing start age	<1 year; 1-2 years; etc
Brushing frequency	≥3 times/d; twice daily; once daily; irregular
Fluoride toothpaste use	Yes; no; unknown
Brushing assistance	Self; helped
Oral hygiene methods	Toothbrush, floss, rinse, etc.
Dental treatment history	
Toothache in past 12 months	Never; sometimes; often; unknown
Dental visits	Yes; no
Time since last visit	<6 months, 6-12 months, or >12 months
Visit reason	Check-up; treatment; prevention
Dental cooperation level	+ + fully cooperative; + cooperative; – + mixed response; – negative; – – completely uncooperative
General anesthesia experience	Yes; no
Oral health knowledge and attitudes	
Oral health knowledge	Correct; incorrect; don’t know
Attitudes toward prevention	Agree; disagree; don’t know
Misconceptions about oral care	True; false; don’t know
Parental burden and psychosocial status	
Time burden	Never to always (5-point scale)
Emotional stress	Never to always (5-point scale)
Social impact	Never to always (5-point scale)
Future concern for child	Never to always (5-point scale)
Disability profile	
Disability type	Vision; hearing; speech; intellectual; autism; physical; multiple; other
Disability severity	Mild; moderate; severe; very severe; unknown
Age at diagnosis	Years
Diagnosing hospital	Hospital name
Medication or comorbidities	Yes; no (specify)
Gastrointestinal and behavioral symptoms	
Stool frequency	Times: week
Stool consistency	Normal; loose; watery
Bloating	Days/week
Abdominal pain	Days/week
Sleep disturbances	Times/week
Feeding behaviors	
Selective eating	Never to always (5-point scale)
Texture or color sensitivity	Never to always (5-point scale)
Food rituals	Never to always (5-point scale)
Disruptive mealtime behaviors	Never to always (5-point scale)
Food refusal behaviors	Never to always (5-point scale)
Oral health examination variables	
Caries type (eg, no caries, crown caries, filled with/without caries), primary vs permanent teeth	A: no caries; B: crown caries; C: filled with caries; D: filled without caries; E: missing due to caries; X: missing for other reasons
Tooth trauma type	Trauma types: enamel crack, enamel fracture, crown-dentine fracture, pulp exposure, avulsion, other
Crown discoloration	+; –
Tooth mobility	+; –
Presence of fistula	+; –
Tooth eruption stage	One-third; two-thirds; full
Presence of supernumerary teeth	Yes; no
Presence of fissure sealant	Yes; no
Presence and type of dental fillings	Yes; no (type noted)
Missing teeth due to caries or other reasons	Yes; no (reason noted)
Exposure of pulp in traumatic crown fractures	Yes; no
Periodontal condition measured using plaque index and gingival index	Clinical description

### Data Collection

Data will be collected at baseline and at the 12-month follow-up according to prespecified study-specific procedures. Trained dental professionals will perform oral examinations. They will use the DMFT index [25]. Examiners also record periodontal status, including plaque index and gingival index, as well as tooth eruption and trauma [26]. At the same time, parents and teachers will complete structured KAP questionnaires. All paper records will be double-entered to reduce errors. Monitors will oversee the process to ensure protocol adherence and data accuracy.

### Statistical Analysis

All data will be entered into a centralized electronic data capture system with built-in logic checks to ensure completeness and internal consistency. Statistical analyses will be conducted using Stata (version 18.0; StataCorp LLC). Descriptive statistics will first be generated to summarize baseline characteristics of participants in both intervention and control groups, including means, SDs, proportions, and frequency distributions.

Comparative analyses between groups will be conducted on an intention-to-treat basis. Participants will be analyzed according to their randomized group, regardless of receipt of FV. For descriptive and unadjusted comparisons between groups, chi-square tests will be used for categorical outcomes (eg, caries prevalence). For continuous variables, independent-samples *t* tests will be used for normally distributed data, and nonparametric tests (eg, Mann-Whitney *U* test) will be applied for skewed distributions. Paired comparisons (eg, baseline vs 12-month follow-up) within groups will use paired *t* tests or Wilcoxon signed-rank tests as appropriate.

To evaluate the effectiveness of the intervention while accounting for potential confounders and cluster randomization in the primary analysis, mixed-effects logistic regression will be used for binary outcomes, such as caries presence, and mixed-effects linear regression for continuous outcomes, such as the number of decayed teeth [27]. Models will adjust for relevant covariates, including age, sex, socioeconomic status, and baseline oral health status. Multiple imputation with chained equations will be applied to handle missing covariate data. Cluster effects will be accounted for using robust SEs or multilevel modeling, as appropriate. Effect estimates will be presented as adjusted odds ratios or mean differences with 95% CIs. All hypothesis testing will be 2-sided, with a significance level set at *P*<.05. Sensitivity analyses, including subgroup analyses by economic region, will be conducted to assess the robustness of the findings.

### Ethical Considerations

This study has been approved by the institutional review board of Sun Yat-sen University (approval 2025‐065) and will be conducted in accordance with the Declaration of Helsinki and relevant national ethical guidelines. Prior to participation, detailed information about the study objectives, procedures, potential risks, and benefits will be provided to all participants and their legal guardians. Informed consent will be obtained from the guardians of participating students younger than 18 years. For students aged 18 years or older, informed consent will be obtained from either the participant or a legal guardian with participant assent [28,29] (Multimedia Appendix 1).

Although FV is considered safe and adverse events are uncommon, all potential adverse reactions will be monitored and recorded [30]. Any adverse reactions will be managed promptly, with medical referral and ethics reporting as required. Participation in the study is voluntary, and individuals may withdraw at any time without penalty. All personal data will be anonymized and stored securely in password-protected systems accessible only to authorized research personnel. Only deidentified data will be used for analysis and reporting to ensure confidentiality.

## Results

This trial was funded by the Department of Education of Guangdong Province (grant YCKJ-2025‐30). Participant recruitment and school selection are expected to be completed by June 2025. Baseline data, including oral health examinations and questionnaires, were collected during the spring semester of 2025. Following baseline assessment, the intervention group received the first professional application of FV and tailored oral health education materials for parents and teachers. A second round of FV application will be delivered 6 months after the first intervention. The control group will continue with standard annual dental examinations without fluoride application. An overview of the study is shown in Figure 2. Data collection is scheduled to conclude in June 2026. Data analysis will begin after the completion of data collection. At the time of protocol preparation, school selection (n=12) was finalized, with 5 (41.7%) control schools and 7 (58.3%) intervention schools enrolled across 3 representative cities, and more than 1100 students had been recruited. Descriptive and analytic results are expected to be published in 2027.

## Discussion

### Hypothesized Findings

Biannual FV application, integrated with tailored oral health education for parents and teachers, is anticipated to be an effective intervention for caries control, significantly reducing the prevalence of dental caries among students in special education schools in Guangdong Province. We anticipate a significantly lower incidence of new caries and a higher rate of oral hygiene improvement—as measured by plaque and gingival indices—in the intervention group. Furthermore, the intervention is expected to significantly improve parental and teacher health literacy and oral health–related KAP scores. This study integrates professional clinical prevention with school-based education, aiming to generate critical evidence to inform national oral health strategies and policies for children with disabilities in China.

### Comparison to Prior Work

Although the efficacy of FV is well-documented in general pediatric populations, research specifically targeting children with disabilities in special education settings remains limited [6-9]. Previous studies in Asia have highlighted significant oral health inequities, noting that students with disabilities experience higher rates of dental caries than their peers without disabilities [1]. However, most prior work has been observational, focusing on the prevalence of oral diseases rather than evaluating systematic interventions within the school infrastructure [31-33].

### Strengths and Limitations

This study uses a stratified cluster RCT design, enhancing internal validity and allowing for robust comparisons across different economic regions. The FV intervention is evidence-based and feasibly integrated into the school setting, facilitating scalable and routine implementation for high-risk populations. Standardized assessment tools and trained personnel are used for oral health examinations and data collection, ensuring measurement consistency and data quality.

Despite these strengths, several limitations should be considered. As the study is conducted within schools, findings may not be generalizable to children with disabilities who are not enrolled in special education institutions.

### Future Directions

Future research may focus on the long-term sustainability of school-based fluoride programs beyond 12 months to assess whether caries prevention persists. Subsequent studies could also incorporate a cost-effectiveness analysis to help health authorities justify integrating FV into routine school health services.

### Dissemination Plan

Findings from this study will be disseminated through peer-reviewed scientific journals, presentations at national and international conferences, and briefings to policymakers and stakeholders in the health and education sectors. In addition, summary results will be shared with participating schools and local health authorities to inform the development of future oral health programs targeting children with disabilities.

### Conclusions

By using a stratified cluster randomized design, the study will provide evidence on the effectiveness of integrating professional FV applications into the special education school environment. If the hypothesized reduction in caries and improvement in health literacy is confirmed, this model could serve as a blueprint for national health authorities to implement scalable, cost-effective oral health programs.

## Supplementary material

10.2196/93889 Multimedia Appendix 1 Trial monitoring plan and procedures for assessing protocol adherence and data quality.

10.2196/93889 Checklist 1 SPIRIT checklist.

## Abbreviations

DMFT: decayed, missing, and filled teeth
FV: fluoride varnish
KAP: knowledge, attitudes, and practices
RCT: randomized controlled trial
