# Supplementary material for: Oral Health Promotion Among Students in Special Education Schools: Protocol for a Multicity Cluster Randomized Controlled Trial
Source: JMIR Res Protoc. 2026 Jul 3;15:e93889. doi: 10.2196/93889 (PMC13331069; doi:10.2196/93889)
Supplement: Multimedia Appendix 1 [file resprot-v15-e93889-s001.docx]

**Appendix 1. External monitoring of the trial**

Suitably qualified external monitors, appointed by the trial sponsor, will oversee the conduct of the study. Monitors will help to ensure that the data collected are accurate and complete and that investigators comply with the study protocol.

Prior to trial initiation, monitors will confirm that all of the study sites are adequately prepared, that study personnel are properly trained and fully familiar with trial procedures, and that a sufficient number of eligible students can be recruited.

During the trial, monitors will ensure that informed consent has been obtained from all participants prior to enrolment. They will track participant recruitment and monitor overall trial progress. Monitors will also verify that investigators are using the most recent versions of all trial-related documents. In addition, monitors will assess adherence to the fluoride varnish (FV) intervention, including whether applications are performed by qualified personnel according to the scheduled timeline. They will verify consistency between reported data and field practices to ensure intervention fidelity.

They will verify that all electronic data entries are complete and consistent with the original source documents (e.g., paper questionnaires). Any corrections or amendments to the data will be reviewed to ensure they are properly documented, dated, and signed by the responsible investigators.

Monitors will also ensure that data is stored securely and in compliance with confidentiality requirements. Monitoring visits will be documented in reports submitted to the trial sponsor, detailing the date, location, scope, observations, protocol deviations (if any), conclusions, and corrective actions taken.

**Appendix 2. Informed consent form**

*Intervention group*

Dear Parent/Guardian,

Greetings!

We are healthcare professionals from the Affiliated Stomatological Hospital of Guangzhou Medical University, the Guangzhou Center for Disease Control and Prevention, and the School of Public Health, Sun Yat-sen University. In order to comprehensively understand the oral health status and care needs of children in special education schools in Guangdong Province, scientifically evaluate the effectiveness of integrated interventions, and provide evidence for relevant policy-making, we are currently carrying out the Oral Health Promotion Program for Students in Special Education Schools.

As part of this program, your child will receive a free oral health examination and fluoride varnish application to prevent dental caries (tooth decay). Fluoride varnish is a commonly used preventive measure in which a fluoride-containing substance is applied to the tooth surface. It is suitable for children and adolescents, working through the following mechanisms:

- Promoting tooth remineralization and strengthening enamel resistance;

- Inhibiting bacterial metabolism, reducing acid production, and lowering the risk of tooth demineralization.

These actions help effectively prevent dental caries. Both the oral health examination and fluoride application are public health services provided free of charge, and will not cause any harm or discomfort to your child. After the examination, we will provide you with a report of your child’s oral health status, helping parents and teachers better monitor and support the child's oral health. All personal information collected will be kept strictly confidential.

If you agree to have your child participate in this program, please assist us by completing the following:

(1) Fill out the Children’s Oral Health Survey;

(2) Consent to your child receiving an oral health examination;

(3) Consent to your child receiving free fluoride varnish for caries prevention.

We sincerely thank you for your support and cooperation!

Oral Health Promotion Program Team for Students in Special Education Schools, Guangdong Province

1. Informed Consent

○ I have read and understood the above information, and I voluntarily consent to my child participating in the oral health survey, oral examination, and fluoride varnish application.

○ I have read and understood the above information, and I voluntarily consent to my child participating in the oral health survey and oral examination, but not the fluoride varnish application.

○ I do not consent to participation.

*Control Group*

Dear Parent/Guardian,

Greetings!

We are healthcare professionals from the Affiliated Stomatological Hospital of Guangzhou Medical University, the Guangzhou Center for Disease Control and Prevention, and the School of Public Health, Sun Yat-sen University. In order to comprehensively understand the oral health status and care needs of children in special education schools in Guangdong Province, scientifically evaluate the effectiveness of integrated interventions, and provide evidence for relevant policy-making, we are currently carrying out the Oral Health Promotion Program for Students in Special Education Schools.

As part of this program, your child will receive a free oral health examination. This examination is publicly funded and provided free of charge, and it will not cause any harm or discomfort to your child. After the examination, we will provide you with a report on your child’s oral health status, helping parents and teachers better manage and monitor their oral health. All personal information collected during the survey will be kept strictly confidential.

If you agree to have your child participate in this program, please assist us by completing the following:

(1) Fill out the Children’s Oral Health Survey;

(2) Consent to your child receiving an oral health examination.

We sincerely thank you for your support and cooperation!

Oral Health Promotion Program Team for Students in Special Education Schools, Guangdong Province

1. Informed Consent

○ I have read and understood the above information, and I voluntarily consent to my child participating in the oral health survey and oral examination.

○ I do not consent to participate
